# The Practitioner’s Hand-Book of Treatment

**Published:** 1877-07

**Authors:** 


*The Practitioner's Hand-Book of Treatment; Or the Principles of Therapeutics.* By J. Milner Fothergill, M. D., Member of the Royal College of Physicians of London; Assistant Physician to the City of London Hospital for diseases of the Chest, Victoria Park, etc. Philadelphia: H. C. Lea. 8vo. pp. 575.

The author of this work shows that he is thoroughly familiar with the current literature of the profession in matters of physiology, pathology and therapeutics. The names of very many authorities are presented in connection with their views on subjects they have specially investigated. He has carefully observed the varying causation and conditions of disease, and advocates the peculiar treatment indicated therein. The book is interesting, to every careful practitioner for the excellent manner and condensed form in which nearly all the recent views and facts in therapeutics are presented. One can here review in a brief time interesting facts which can be gathered only by extensive reading. It does not appear that the author relies upon his personal experience in the methods of treatment quite so much as would be expected. His statements are mainly such as are upheld by the general experience of physicians; in doubtful and changing methods of treatment, he presents facts of the physiological action of remedies advocated, and appeals to the profession for recognition of their value. He is discreet in the size of his doses, very intelligent in his selection, not so careful as to the palatability of his formula. The reader is very pleasantly, and frequently reminded of how near the author's views coincide with his own, and very many useful ideas he will find called to his attention here for the first time.

We think he does not rightly interpret the sentiment of the best authors and practitioners of the United States to-day, upon the matter of depressants in the acute febrile stages of disease, when he quotes Ringer;—"Aconite is to be most esteemed for its power, little less than marvelous, of controlling inflammation, and subduing the accompanying fever. It will sometimes at once cut short an inflammation. It will not remove the products of inflammation, but by controlling the inflammation, it prevents their formation so saving the tissues from further injury."

As examples in which antimony is to be given, he cites whitlow and tonsillitis, also pleuritis from tubercle; his dose however, is far below that which was in common vogue twenty years ago. The remedy has been nearly abolished of late. It is valuable to compare present editions with former ones of our best authorities and notice which way progress is tending. The sensible views which were advocated in Flint's Practice years since, have of late been almost unqualifiedly adopted by our able writers on children's diseases, and it would take very positive experimental evidence to conteract this tendency here.

In reference to the value of digitalis, the following is quoted from his chapter on the circulatory system. "In no class of diseases has there been so much improvement wrought in treatment by physiological research as in the diseases of the heart." "The correct understanding of the action of digitalis, of the class of cases to which it is suited, and those where its use is contraindicated from a subject upon which every practitioner and every student ought to have definite and distinct ideas; that is, if he wish to hold his own in the present arduous struggle for existence." W.

